# Lessons learnt from imported cases and onward transmission of Lassa fever in Europe support broader management of viral haemorrhagic fevers

**DOI:** 10.2807/1560-7917.ES.2017.22.39.17-00661

**Published:** 2017-09-28

**Authors:** Roger Hewson

**Affiliations:** 1 Public Health England, Porton Down, United Kingdom; 2 Faculty of Infectious Tropical Disease, London School of Hygiene & Tropical Medicine, United Kingdom

**Keywords:** Lassa fever, viral haemorrhagic fever

Viral haemorrhagic fevers (VHFs) are severe and life-threatening diseases that have been reported in parts of Africa, South America, the Middle East, Central Asia and Europe. They are capable of causing long-term and slow burning epidemics, which can interrupt normal life, trade and impact the social structure of a community [1]. The known viruses responsible for VHFs are not genetically related and are classified in disparate virus families; they include members of the *Arenviridae*, *Flaviviridae*, *Filoviridae*, *Nairoviridae* and *Phenuviridae* (Table). They circulate in animal reservoirs and sometimes arthropod vectors; humans are not part of their natural life cycle, but incidental infection and introduction into the human population often results in an outbreak. VHFs are described as hazard or risk group 4 pathogens [2,3] and warrant maximum laboratory containment facilities and working precautions i.e. containment or biosafety level 4.

**Table:** Viruses known to cause viral haemorrhagic fever in humans

Virus taxonomy VHF	Reservoir / vector	Geographic location / risk areas	Disease	Incubation period (days)	Case fatality rate^a ^(%)	Reference
***Arenaviridae***						
Chapare mammarenavirus	Rodents^b^	South America / Bolivia	Chapare HF	7–14	70^a^	[13]
Guanarito mammarenavirus	Rodents *Zygodontomys brevicauda*	South America / Venezuela	Venezuelan HF	6–14	36	[14]
Junín mammarenavirus	Rodents *Calomys musculinus*	South America / Argentina	Argentinian HF	7–14	3–15	[15]
Lassa mammarenavirus	Rodents *Mastomys natalensis*	West Africa / Sub-Saharan Africa	Lassa fever	5–6	1	[16]
Lujo mammarenavirus	Rodents^b^	Sub-Saharan Africa	Lujo HF	7–14	80	[17]
Machupo mammarenavirus	Rodents *Calomys callosus*	South America / Bolivia	Bolivian HF	7–14	25	[18]
Sabiá mammarenavirus	Rodents^b^	South America/ Brazil	Brazilian HF	7–14	50^a^	[19]
***Flaviviridae***						
Yellow fever virus	Monkeys / mosquitoes	South America and Africa	Yellow fever	3–6	20–50	[20]
***Filoviridae***						
Ebolavirus	Bats^b^	Sub-Saharan Africa	Ebola HF	2–21	50–80	[21]
Marburg virus	Fruit bats *Rousettus aegyptii*	Sub-Saharan Africa	Marburg HF	2–21	80	[21]
***Nairoviridae***						
Crimean-Congo haemorrhagic fever orthonairovirus	Ticks / rodents and ruminants	Asia, Africa Middle East Europe	Crimean Congo HF	1–3	5–50	[22]
***Phenuiviridae***						
Rift Valley fever virus phlebovirus	Livestock ruminants / mosquitoes	Sub-Saharan Africa	Rift Valley fever	2–6	1	[23]
Severe fever with thrombocytopenia syndrome phlebovirus	Deer / ticks	China, South Korea Japan	Severe fever with thrombocytopenia syndrome	7–14	12–40	[24]

HF: haemorrhagic fever; VHF: viral haemorrhagic fever.

^a^ Some case fatality rates are based on low numbers of cases.

^b^ Animal reservoirs suspected but not proven.

VHFs are of particular public health importance because they can spread within hospital and community settings; they have a high case fatality rate if left untreated; they are difficult to recognise and detect rapidly; and there is no specific treatment. In Europe, environmental conditions do not support the natural animal reservoirs or vectors of most of the haemorrhagic fever viruses, and in consequence the majority of recorded cases of VHF in Europe have been acquired overseas. A notable exception to this rule is the Crimean-Congo haemorrhagic fever virus which occasionally infects humans. It circulates unobtrusively between mammals and ticks in southern parts of west and eastern Europe [4]. Autochthonous cases of other VHFs have infrequently occurred in Europe and, with the exception of a laboratory worker who sustained a needle-stick injury in the United Kingdom (UK) in 1976 [5], these have been associated with transmission from index cases to healthcare workers [6,7]. Such incidents attract interest and ultimately help our understanding of disease transmission, leading to improvements in the risk assessment and management of VHFs. Over the past decade, Europe has faced a total of 21 different incursions of VHF [8-14].

In this issue of *Eurosurveillance* two articles by Lehmann et al. and Ehlkes et al. [15,16] provide further insight to the VHF community by detailing the most recent importation of Lassa fever (LF) into Germany in 2016 and its onward transmission to an undertaker. The incident highlights difficulties and delays in the diagnosis of the index case, principally because the country where the infection was acquired – Togo – had not previously been known as an endemic area of Lassa fever virus (LASV). The index case died soon after admission and a post-mortem was performed. Although this did not disclose the immediate cause of death, liver sections displayed classic features of VHF, and LASV was subsequently detected in a laboratory test. Unfortunately, this diagnosis occurred after the patient, the body and tissue samples had come into contact with healthcare workers and others on multiple occasions in the absence of specific precautions required for a hazard group 4 pathogen. Extensive effort in contact tracing, risk categorisation, interviewing and reporting then ensued. Symptom monitoring in contacts was complicated by a peak in activity of the influenza season and plans to avoid the unnecessary triggering of enhanced control measures such as isolation or quarantine were developed with an underpinning and basic scientific knowledge of LASV and human disease. An undertaker, who had contact with the body 6 days after death, was identified at increased risk following interview and while an early PCR test for LASV was negative, he tested positive 12 days post exposure. The undertaker was subsequently treated with ribavirin and made a full recovery; it is unclear how he became infected. None of the other 76 contacts who were followed up in the two concerned German federal states, North-Rhine-Westphalia and Rhineland-Palatinate, were positive.

Following an international alert after the confirmed diagnosis of the index case, another secondary transmission was diagnosed in a healthcare professional who had cared for the index case in Togo. This patient was subsequently medevacked to the Unites States and made a full recovery following treatment.

The rodent reservoir of LASV - the Natal multimammate rat *Mastomys natalensis* - is not supported by environmental conditions in Europe and LF does not pose a significant public health threat in this continent. Nevertheless, LF cases are the most commonly imported of the VHFs into Europe, with 21 traveller-related incidents since the virus was discovered in 1969; interestingly, 13 of these were to the UK [17]. Given the long-standing deep-seated links with Europe and the role of European workers in humanitarian support in West Africa, LF cases will continue to be imported into Europe. Each incident places a substantial demand on clinical, laboratory and public health resources [18]. It is fitting therefore that scientific knowledge is continually developed, including a better understanding of similar emerging viruses [19-30]. Lessons learnt should be continually distilled into appropriate guidance so that future VHF incidents can be effectively managed and rapidly controlled as also pointed out by Ehlkes et al. and Lehmann et al..

From a global perspective, the ideal way to prevent imported infections would be to control the disease in West Africa. Rodent control and the reduction of contact between humans and rodents and their excreta will help to prevent infection. Other control measures such as vaccination may offer a more sustainable solution. Indeed LF has been prioritised for research and development in public health emergency contexts by the World Health Organization [31] and several international vaccine strategies are underway.
